# Detection of nucleophosmin and FMS-like tyrosine kinase-3 gene mutations in acute myeloid leukemia

**DOI:** 10.4103/0256-4947.75778

**Published:** 2011

**Authors:** Vahid Pazhakh, Farhad Zaker, Kamran Alimoghaddam, Farzaneh Atashrazm

**Affiliations:** a From the Cellular and Molecular Research Center, Iran University of Medical Sciences (IUMS), Tehran, Iran; b From the Hematology-Oncology and Stem Cell Transplantation Research Center, Shariati Hospital, Tehran, Iran

## Abstract

**BACKGROUND AND OBJECTIVES:**

Nucleophosmin gene mutations are frequently reported in acute myeloid leukemia (AML) patients with normal karyotype, which is also frequently associated with internal tandem duplication mutations in the FMS-like tyrosine kinase-3 gene. We sought to detect the nucleophosmin and FMS-like tyrosine kinase-3 (FLT3) internal tandem duplication (ITD) mutations among Iranian patients with AML and to assess the relationship between these mutations and the subtypes of the disease.

**DESIGN AND SETTING:**

Cross-sectional study of patients referred during 2007 through 2009.

**PATIENTS AND METHODS:**

Bone marrow and peripheral blood samples of 131 AML patients were randomly collected at the time of diagnosis and prior to treatment and the DNA extracted. After amplifying the nucleophosmin and FLT3 gene regions, positive cases were screened by conformation-sensitive gel electrophoresis and agarose gel electrophoresis techniques.

**RESULTS:**

Of 131 patients, 23 (17.5%) (0.95% CI=0.107-0.244) had nucleophosmin gene mutations. The highest frequency of such mutations was found among the subtypes of M4 (30.4%), M3 (21.7%) and M5 (17.4%). There was a high frequency of these mutations in the M3 subtype as well as a high frequency of allele D in all subtypes. Also, 21 (16.0%) samples (0.95% CI=0.092-0.229) had FLT3/ITD mutation, of which 8 samples had mutant nucleophosmin (8 of 23, 35%), and another 13 samples had wild-type nucleophosmin gene (13 of 108, 12%). There was a high degree of association between the occurrence of nucleophosmin and FLT3/ITD mutations (*P*=.012).

**CONCLUSION:**

Our data showed a high frequency of NPM1 mutations in the monocytic subtypes of AML, as well as a high degree of association between the occurrence of NPM1 and FLT3/ITD mutations.

The development of molecular analysis in recent years has provided novel and important indicators for the prognosis of acute myeloid leukemia (AML). Internal tandem duplications (ITDs) in the FMS-like tyrosine kinase-3 (FLT3) gene,^1^,^2^ partial tandem duplications in the mixed lineage leukemia gene (MLL)^3^,^4^ and enhanced expression of replication factor ectopic virus integration site 1^5^ are all indicators of poor prognosis. In turn, mutation in replication factor CCAAT/enhancer-banding protein α (CEBPA) is associated with good response to therapy.^6^,^7^ With current karyotyping methods, no chromosomal abnormality is found in 40% to 50% of AML patients, and differentiation between different prognostic subtypes in this group of patients by present approaches to molecular genetics seems to be of great importance.^4^,^8^,^9^ The majority of these cases are characterized as “AML not otherwise characterized” by the WHO categorization.^10^

The findings of a recent study conducted by Falini et al11 on mutations in exon 12 of the nucleophosmin (NPM1/B23) coding gene in chromosomal location 5q35 showed that NPM1 mutations occur in 35.2% of primary AML cases and these mutations are associated with such features as having a high incidence in all morphologic subtypes of AML (the highest frequency was seen in monocytic leukemias), an absence of hematopoietic stem cell markers like CD34 and CD133, a normal karyotype and responsiveness to chemotherapy.

NPM1 is a phosphoprotein with high expression in proliferating cells and a member of the nucleoplasmin (NPM) family of proteins that is normally localized in nucleolus.^12^,^13^ Several functions have already been described for NPM1 protein, including binding to nucleic acids,^14^ regulation of centrosome duplication^15^ and also ribosomal function.^16^ Furthermore, NPM1 binds to several proteins, like p53 and proteins that react to and regulate p53 (like Rb,17 p19ARF18 and HDM219). The high frequency of NPM1 mutations in normal-karyotype AMLs and the fact that cytoplasmic NPM1 fails to carry out its normal functions such as binding to proteins and transferring them lead to the hypothesis that mutations in NPM1 would be a primary event in leukemogenesis.^20^ Therefore, it seems that NPM1 mutations lead to acquiring additional genetic changes in AML leukemic cells.^21^ Moreover, NPM1 mutations often occur in association with FLT3/ITD mutations.^11^ FLT3 is a receptor tyrosine kinase with important roles in hematopoietic stem/progenitor cell survival and proliferation.^22^

The purpose of this study was the assessment of the frequency and sequence of NPM1 mutations among Iranian AML patients, the detection of the frequency of FLT3/ITD mutations in this group and to examine the relationship between these mutations and the FAB (French-American-British classification) subtypes of AML.

## PATIENTS AND METHODS

Bone marrow and peripheral blood samples of 131 AML patients (mean age, 45.9 years; male-to-female ratio, 1.57) (**Table 1**) were randomly taken at the time of diagnosis and before administration of chemotherapy from the Iranian Blood Transfusion Organization (IBTO) and Hematology-Oncology and Stem Cell Transplantation Research Center of Shariati Hospital (Tehran, Iran). The Medical Ethics Committee of the Iran University of Medical Sciences (IUMS) approved the study (project number P-516), and written informed consent of all patients who participated was obtained.

**Table 1:** NPM1 and FLT3/ITD mutations in patients with AML.

FAB subtypes	All samples (%)	NPM1+ (%)	FLT3 ITD+ (%)	NPM1+/FLT3 ITD+(%)
M0	3 (2.2)	1 (4.34)	0	0
M1	17 (12.97)	1 (4.34)	2 (9.52)	0
M2	40 (30.53)	4 (17.39)	5 (23.80)	1 (12.5)
M3	30 (22.9)	4 (17.39)	7 (33.33)	2 (25)
M3v	1 (0.76)	1 (4.34)	1 (4.76)	0
M4	29 (22.13)	7 (30.43)	5 (23.8)	3 (37.5)
M5	9 (6.87)	4 (17.39)	0	2 (25)
M6	1 (0.76)	1 (4.34)	1 (4.76)	0
M7	1 (0.76)	0	0	0
Total	131	23	21	8

**Figure 1:**
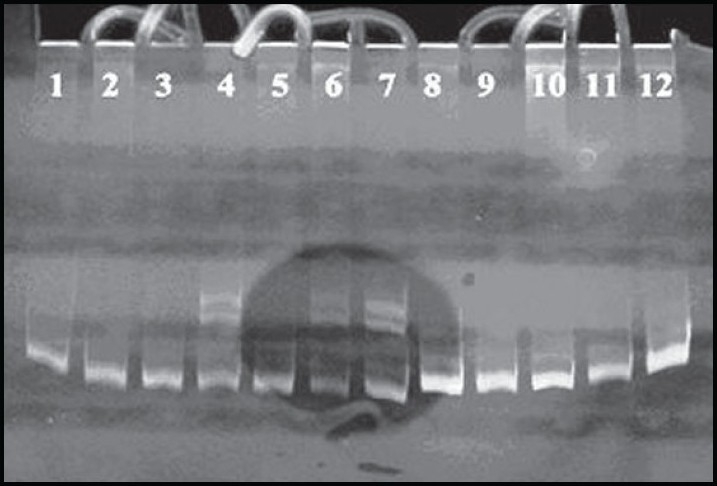
Mutant NPM1 on CSGE gel in AML patients. The upper bands in samples 4, 6 and 7 are related to heteroduplex formation between wild-type and mutant alleles of NPM1 gene.

Blasts and mononuclear cells were purified by Ficoll-Hypaque (Pharmacia LKB, Uppsala, Sweden) centrifugation and their DNA was extracted by the standard method.^23^ Diagnoses were done through morphologic, cytochemical and immunophenotypic assessments by flow cytometry and the patients classified according to FAB criteria. To screen the NPM1 mutations, polymerase chain reaction (PCR) amplification of NPM1 exon 12 was carried out using primers NPM1-F (5’-TTAACTCTCTGGTGGTAGAATGAA-3’) and NPM1-R (5’-CAAGACTATTTGCCATTCCTAAC-3’), as described in previous studies.^11^ The total reaction volume of 50 μL contained approximately 100 ng DNA, 10 pmol of each primer, deoxynucleoside triphosphates (dNTPs, 10 mM each), 2.5 U Prime Taq polymerase and reaction buffer (GENET BIO, Korea) and ddH_2_ O. Samples were amplified using the following PCR conditions: 95°C for 5 minutes; 40 cycles of 94°C for 30 seconds; 55°C for 1 minute; 72°C for 1 minute and finally 72°C for 7 minutes (Techne TC-512).^24^ PCR products were visualized by agarose gel electrophoresis with a suitable gene ruler (Gene Ruler 50-bp DNA ladder) to confirm the amplification of fragment of concern (560 bp). Samples were then put in a conformation-sensitive gel electrophoresis (CSGE) program at 95°C for 5 minutes (denaturizing); 65°C for 30 minutes (annealing). To determinate any probable homozygote mutations, samples were mixed in pairs and run under the CSGE program. Then, all the products were subjected to electrophoresis under 350 V for 4 hours on 10% CSGE gel. Finally, the CSGE gel was stained with ethidium bromide and exposed to UV light to screen the samples carrying mutation base on different electrophoretic bands (**Figure 1**).^25^

PCR products screened by CSGE technique were purified (AccuPrep PCR purification kit, BioNEER, Korea) and then directly sequenced using NPM1-F and NPM1-R primers (3730XL DNA analyzer, Labège, France).

**Figure 2:**
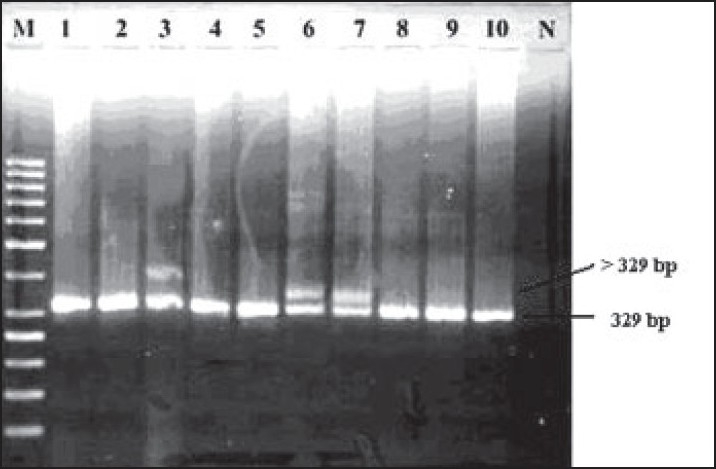
Mutant FLT3/ITD on agarose gel in AML patients. The lower bands are related to 329-bp fragment (wild-type allele of FLT3 gene); the upper band in samples 3, 6 and 7 is related to fragment larger than 329 bp which is mutant. (The upper band size depends on numbers of internal tandem duplications in FLT3 gene.) M: DNA marker, N: negative control.

All DNA samples were then amplified with a PCR step specific to FLT3 gene using primers FLT3/ITD-F (5’-GCAATTTAGGTATGAAAGCCAGC-3’) and FLT3/ITD-R (5’-CTTTCAGCATTTTGACGGCAACC-3’), and their PCR products were subjected to electrophoresis on 2.5% agarose gel (100 V for 1 hour) to screen FLT3/ITD mutations (**Figure 2**).^26^ The Fisher exact test was applied to estimate the association between NPM1 and FLT3/ITD mutations.

## RESULTS

**Figure 3:**
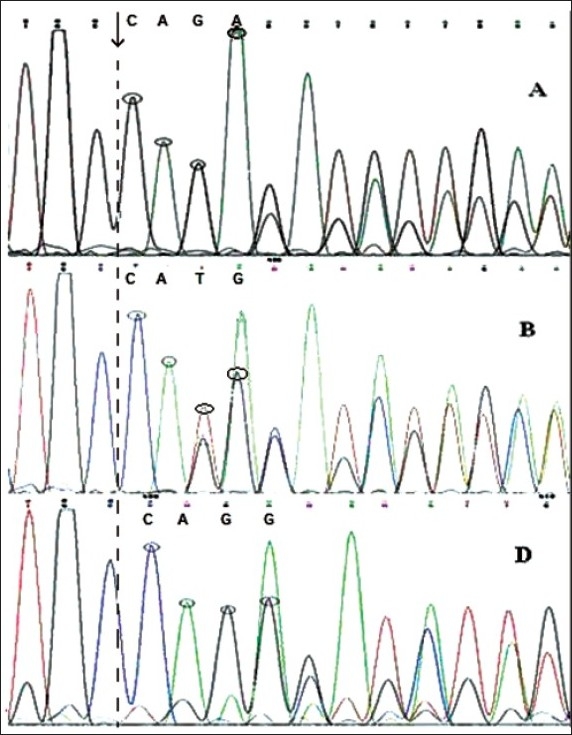
Sequence results for NPM1 mutations. (a) Sequence result in a patient with allele A of mutant NPM1 in reverse direction; (b) sequence result in a patient with allele B of mutant NPM1 in reverse direction; (c[S1]) sequence result in a patient with allele D of mutant NPM1 in reverse direction.

Among the 131 AML patients who participated in this study,^23^ (17.55%) patients (0.95% CI=0.107-0.244) had NPM1 mutations. The presence of these mutations was confirmed using the sequencing technique. Table 1 shows the FAB subtypes among the 23 cases with NPM1 and FLT3/ITD mutations. Among the 23 patients with NPM1 mutation, 14 (60.8%) patients had the mutant allele A, 5 (21.7%) patients had allele D, and 4 (17.4%) patients had allele B. Mutations of NPM1 were heterozygous in all 23 screened patients (**Figure 3, Table 2**). Of 131 patients, 21 (16.03%) patients (0.95% CI=0.092-0.229) had FLT3/ITD mutations, among whom 2 patients had M1 (9.52%); 5 patients, M2 (23.8%); 7 patients, M3 (33.33%); 1 patient, M3v (4.76%); 5 patients, M4 (23.8%); and 1 patient, M6-AML (4.76%). Among AML patients with FLT3/ITD mutations, 8 patients had mutant NPM1 (8 out of 23, 35%), while the other 13 patients had wild-type NPM1 gene (13 of 108, 12%) (**Table 1**).

## DISCUSSION

The high frequency of NPM1 mutations among AML patients and their prognostic value underscore the importance of screening for these mutations. Different methods have been described so far to screen and assess NPM1 mutations. These include use of monoclonal antibodies and immunohistochemistry,^11^ denaturing high-performance liquid chromatography (DHPLC),27 capillary electrophoresis of multiplex PCR products,^28^ and also fluorescence resonance energy transfer (FRET).^29^ In our study, the conformation-sensitive gel electrophoresis (CSGE) technique was used to screen AML patients with NPM1 mutations. The advantage of low cost, easy application and the existence of reports implying adequate sensitivity of this technique in mutation detection,^30^ as well as the absence of any existing report describing screening NPM1 mutations by CSGE–all convinced the researcher to use this technique.

In the study by Falini et al,^11^ 38% of AML patients had normal karyotype, among whom 61.7% had NPM1 mutations. The total frequency of NPM1 mutations among all AML cases in this study was reported to be 35.2%. In the study by Thiede et al,^31^ the frequency of NPM1 mutations among all AML cases was reported as 27.4%. In the study by Zhang et al,^32^ 14.3% of AML patients with normal karyotype were reported to have a mutant NPM1. The other results were different: 24.9% in all AML cases,^33^ 74.3% in normal-karyotype AMLs^34^ or 52.9% in normal-karyotype AMLs.^10^ In fact, in spite of relative differences in frequencies of these mutations, all studies imply a higher frequency of NPM1 mutations in AML cases with normal karyotype.

In our study, NPM1 gene mutations were detected in 17.5% of AML cases. Due to the unavailability of karyotype information for these patients, assessments of the frequency of NPM1 mutations among AML cases with normal or abnormal karyotype in our study was not possible. However, on the basis of previous reports on the higher frequency of cytogenetic abnormalities among Iranian AML patients,^35^ it can be said that it is not unlikely that the above-stated fact has accounted for the lower frequency of NPM1 mutations in our results. Falini et al^11^ reported NPM1 mutations in AML subtypes based on FAB classification in all subtypes except M3, M4Eo and M7. In the study by Falini et al, the highest frequency was in M5b (87%), whereas the lowest was in M0 (13.6%). In the study by Verhaak et al,^20^ the emphasis was on the high rate of NPM1 mutations in M5 and M6 subtypes, and the lower frequency in other subtypes, including M3, but they did not report mutations in M0. In the study by Döhner et al,^24^ the highest frequency of NPM1 mutations was reported in M4 and M5. Thiede et al^31^ reported on NPM1 mutations mainly in M2, M5a and M5b, but not in the M3 subtype. No mutation was reported in the M3 subtype in the study by Suzuki et al.^33^

Among the interesting points in our results was the high frequency of NPM1 mutations in the M3 (and M3v) subtype (21.7% of all mutations that had been detected), while most of the previous studies have reported a lower frequency of NPM1 mutations in this group of patients. However, the finding of highest frequency of these mutations in the monocytic subtypes of FAB (M4+M5=47.8%) is completely corroborated by other studies.

Several studies emphasize the high frequency of NPM1 mutations in middle-aged adults or more elderly individuals (average age, 58 years)^33^ and the rare incidence of mutations in individuals younger than 35 years.^10^,^33^ In our data, the age pattern for NPM1 mutations in the Iranian context seems similar to that revealed by previous investigations. In other words, there seems to be a direct relationship between increase in age and the NPM1 mutation incidence in AML patients.

Three major types of mutations in the form of insertion/ deletion in the c-terminal region of exon 12 in NPM1 gene (in position 960) have been detected: (1) Insertion of 4 nucleotides, like alleles A, B, C and D, which have high frequency. Alleles A and B have been reported to account for 78% and 12% of all NPM1 mutations, respectively.^36^ (2) Deletion of 4 or 5 nucleotides and insertion of 9 new nucleotides in that position, like alleles E and F. (3) Deletion of 9 nucleotides and insertion of 14 new nucleotides. All these mutations finally lead to 4 to 5 nucleotides elongation in this region compared with the wild-type allele and a frame shift mutation in C-terminal of the protein (**Figure 3, Table 2**). In all of these situations, at least one of two tryptophan (W) residues in positions 288 and 290 (WQWRKSL), which are necessary for the nucleolar localization of NPM1, shift into other amino acids.^31^ Of 23 patients who had mutant NPM1 in our study,^14^ (60.8%) patients had mutant allele A; 5 (21.7%), allele D; and 4 (17.4%), allele B. Our results showed a high frequency of allele D of the mutant NPM1 gene among Iranian AML patients.

**Table 2:** Inserted nucleotides in NPM1 exon 12 mutations.

Wild type (forward sequence) ←	A	C	G	G	T	C	T	C	T	A	G	A	A	C	T	T
Wild type (reverse sequence) →	T	G	C	C	A	G	A	G	A	T	C	T	T	G	A	A
Allele A (forward sequence) ←	A	C	G	G	T	C	T	G	T	C	T	C	T	A	G	A
Allele A (reverse sequence) →	T	G	C	C	A	G	A	C	A	G	A	G	A	T	C	T
Allele B (forward sequence) ←	A	C	G	G	T	A	C	G	T	C	T	C	T	A	G	A
Allele B (reverse sequence) →	T	G	C	C	A	T	G	C	A	G	A	G	A	T	C	T
Allele D (forward sequence) ←	A	C	G	G	T	C	C	G	T	C	T	C	T	A	G	A
Allele D (reverse sequence) →	T	G	C	C	A	G	G	C	A	G	A	G	A	T	C	T

Inserted nucleotides marked with bold characters. Symbols ← and → show direction of nucleotides.

Internal tandem duplication of the FLT3 gene is found in nearly 20% of AML and 5% of myelodysplastic syndrome cases.^22^ A previous study in Iran showed a frequency of 18% of FLT3/ITD mutations among Iranian AML patients, especially of the M3 subtype.^37^ Our results showed 16.0% of all 131 AML patients who participated in our study had FLT3/ITD mutations. The significance of simultaneous assessment of both mutations is due to the fact that most studies show that a favorable prognostic value of NPM1 mutations is only valid in the absence of FLT3/ITD mutations.^10^,^11^,^31^ It has been shown that a NPM1Pos/FLT3-ITDNeg mutations profile represents an independent predictor for a favorable outcome in younger AML patients, especially in those without cytogenetic abnormalities.^10^

In the study by Falini et al,^11^ FLT3/ITD mutations in AML patients with a mutant NPM1 gene occurred twice as often as in AML patients with wild-type NPM1, which suggests a relationship between FLT3/ITD and NPM1 mutations in tumor genesis. In our study, there was a high level of association between mutations in NPM1 and FLT3 (ITD) genes, such that FLT3/ITD mutations had occurred in NPM1-positive AMLs three times more than NPM1-negative AMLs (34.78% vs. 12.03%) (Fisher exact test, *P*=.012). Since we found no false-positive result after sequencing our positive samples (detected by CSGE method), CSGE seems to have enough specificity for screening NPM1 mutations. However, no judgment can be made about the sensitivity of the CSGE method since we had done no sequencing to confirm our results for negative NPM1 mutations. Of course, all major types of NPM1 mutations (alleles A, B and D) were detected by the CSGE method in the current study.

In conclusion, our data showed a high frequency of NPM1 mutations in the monocytic subtypes of AML, as well as a high degree of association between occurrence of NPM1 and FLT3/ITD mutations. Also, the CSGE was found to be a simple and inexpensive test and an acceptable technique to be used in the screening of NPM1 mutations, although further evaluation is needed.
